# Correlation Between Ultrasonographic Features and Histopathological Findings in Breast Lesions

**DOI:** 10.7759/cureus.83699

**Published:** 2025-05-08

**Authors:** Vivek Dhakal, Om B Panta, Sudhir K Yadav, Utkrisht Shah, Ram K Ghimire

**Affiliations:** 1 Department of Radiology, Leighton Hospital, Crewe, GBR; 2 Department of Radiology, Beth Israel Deaconess Medical Center, Harvard Medical School, Boston, USA; 3 Department of Radiology, The First Affiliated Hospital of Dali University, Dali, CHN; 4 Department of Surgery, Nepal Police Hospital, Kathmandu, NPL; 5 Department of Radiology, Nepal Medical College Teaching Hospital, Kathmandu, NPL

**Keywords:** breast, breast malignancy, correlation, histology, ultrasound

## Abstract

Introduction

High-resolution ultrasound has been used successfully to differentiate benign and malignant breast lesions. The purpose of this study was, therefore, to examine the correlation of ultrasound findings, including shape, margin, echotexture, posterior acoustic features, orientation, and calcification, with the histology.

Methods

A retrospective, hospital record-based cross-sectional study was carried out in the Department of Radiology and Imaging. All patients undergoing ultrasound-guided breast biopsies performed from June 2018 to June 2021 were included in the study. Ultrasound images were retrospectively analyzed using the Picture Archiving and Communication System (PACS, GE Healthcare, Chicago, IL, USA). Lesions were analyzed for shape, margin, orientation, echotexture, posterior acoustic features, presence of calcification, and associated features as per the Breast Imaging Reporting and Data System (BI-RADS) Ultrasound Lexicon 2013 (American College of Radiology, Reston, VA, USA). Histopathological findings of the lesions were collected from the Department of Pathology.

Results

A total of 153 biopsies in 153 patients were evaluated. Out of 153 lesions, 119 (77.78%) were benign and 34 (22.22%) were malignant. Fibroadenoma (55, 46.2%) was the most common benign lesion, and invasive carcinoma of no specific type (25, 73.5%) was the most common malignant lesion.

Conclusion

Age, shape, orientation, margins, and calcification were significantly associated with the benign or malignant nature of the lesion. The ultrasound features, such as shape, margin, calcification, and orientation, help to differentiate breast masses as benign or malignant.

## Introduction

Breast cancer is the third most common cancer in Nepal and the second most common malignancy among Nepalese women [1]. Currently, there are no national guidelines for breast cancer screening in Nepal. Furthermore, mammography is not available in most healthcare centres across Nepal.

Differentiating benign from malignant breast lesions is pivotal in the management of breast diseases. Early menopause, late menarche, and genetic predisposition are a few risk factors for breast cancer [2].

Mammography remains the gold standard for breast cancer screening, and advancements like magnetic resonance imaging and molecular testing have further enhanced diagnostic accuracy. These emerging technologies help improve sensitivity and specificity in detecting breast malignancies. However, despite these improvements, accessibility to screening programs remains a significant challenge, particularly in resource-limited settings [3].

In such resource-constrained settings, ultrasound has been shown to be an effective tool for breast cancer detection, especially where mammography is not available. Ultrasound is particularly beneficial in detecting small lesions and in younger patients, as dense breast tissue in these cases can obscure lesions on mammography [4].

Ultrasound assesses multiple features of breast masses, including size, shape, margins, echogenicity, calcifications, and posterior characteristics. Breast lesions can vary in shape - round, oval, irregular, or lobulated - and may have different margins, including smooth, obscured, indistinct, or spiculated edges. Benign lesions are generally round or oval with well-defined margins, whereas malignant lesions are often irregular in shape with poorly defined or spiculated borders, as seen on imaging [5].

Additionally, ultrasound is invaluable in procedures like image-guided fine needle aspiration and biopsy for histopathological assessment. Common causes of benign breast masses include fibrocystic disease, fibroadenoma, cysts, galactocele, and abscesses. Malignant breast disease encompasses various histologic types, including infiltrating and in situ ductal or lobular carcinoma [6].

The purpose of this study was to examine the correlation of ultrasound findings with the histopathological nature of the lesion, whether benign or malignant. Furthermore, establishing a correlation between the two examinations could help in avoiding unnecessary procedures for typically benign lesions or could alert to the urgency of treatment in those cases with higher chances of malignancy based on their imaging characteristics.

## Materials and methods

This study was a retrospective, hospital record-based, cross-sectional study conducted in the Department of Radiology and Imaging of Nepal Mediciti Hospital, Lalitpur, Nepal. All patients who underwent ultrasound-guided breast biopsies between June 2018 and June 2021 were included. Informed consent was taken from each patient. Ethical approval was obtained from the ethics committee of the institution and has an approval number of IRC-RP-2078t79-007. Patients whose biopsy results were inconclusive or whose ultrasound images could not be retrieved from the Picture Archiving and Communication System (PACS, GE Healthcare, Chicago, IL, USA) were excluded from the study.

Ultrasound images were retrospectively analyzed using PACS. Lesions were assessed based on the Breast Imaging Reporting and Data System (BI-RADS) Ultrasound Lexicon 2013 (American College of Radiology, Reston, VA, USA), evaluating characteristics such as shape, margin, orientation, echo pattern, posterior acoustic features, calcifications, and associated findings. The final assessment category was classified into four categories using BI-RADS 2 (benign), BI-RADS 3 (probably benign), BI-RADS 4 (suspicious of malignancy), and BI-RADS 5 (highly suggestive of malignancy). Histopathological findings of the lesions were obtained from the Department of Pathology. These findings were classified as benign for conditions of a non-cancerous nature and malignant for conditions of a cancerous nature.

Data were recorded in a pre-designed proforma and entered into a Microsoft Excel worksheet (Microsoft® Corp., Redmond, WA, USA). Statistical analysis was performed using IBM SPSS Statistics for Windows, Version 24 (Released 2017; IBM Corp., Armonk, New York, United States). Categorical variables were summarised as numbers and percentages, while numerical data were expressed as mean ± standard deviation. The association between ultrasound findings and histopathological results was analyzed using the chi-square test, with Fisher’s exact test applied when the expected cell count was less than five. The results were presented in the form of texts and tables. The level of statistical significance was set at p < 0.05 at a 95% confidence interval.

## Results

A total of 153 biopsies in 153 patients were evaluated. The mean age of the study population was 42.27 ± 12.83 years, with a minimum age of 18 years and a maximum age of 81 years. Among the lesions, 73 (47.7%) were located on the left side, while 80 (52.3%) were on the right side.

Of the 153 lesions, 119 (77.78%) were benign and 34 (22.22%) were malignant. Table 1 shows the histological outcomes for benign lesions, and Table 2 shows the histological outcomes for malignant lesions.

**Table 1:** Histopathological diagnosis of benign nature. IAC: International Academy of Cytology

Benign lesions	Number (N=119)
Benign proliferative lesion (ductal and lobular hyperplasia)	28 (23.5%)
Duct papilloma	2 (1.7%)
Inflammatory breast lesion	14 (11.8%)
Cyst with inspissated content	4 (3.4%)
Benign phylloides	3 (2.5%)
Fat necrosis	2 (1.7%)
Fibroadenoma	55 (46.2%)
Fibrocystic disease	9 (7.6%)
IAC Yokohama System: atypical category	2 (1.7%)

**Table 2:** Histopathological diagnosis of malignant nature.

Malignant lesions	Number (N=34)
Invasive lobular carcinoma	2 (5.9%)
Invasive carcinoma of no specific type	25 (73.5%)
Ductal carcinoma in situ	3 (8.82%)
Suspicious focus of malignancy in a complex sclerosing lesion	2 (5.9%)
Classic adenoid cystic carcinoma	1 (2.9%)
Lymphoproliferative lesion	1 (2.9%)

**Figure 1:**
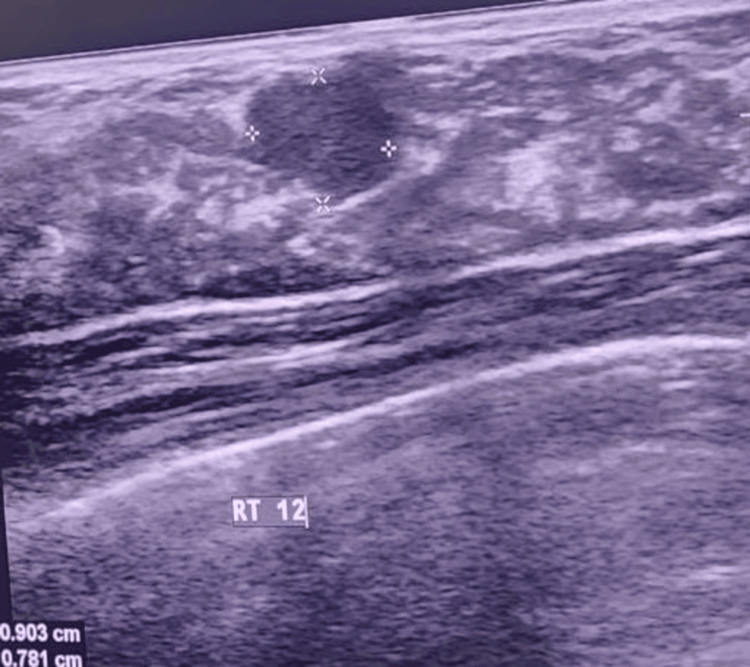
Sonographic image of a fibroadenoma showing a well-defined, hypoechoic mass with an oval shape.

Fibroadenoma was the most common benign lesion (Figure 1), accounting for 55 (46.2%) cases, while invasive carcinoma of no specific type (NST) was the most common malignant lesion, comprising 25 (73.5%) cases. The mean age of patients with benign lesions was 39.37 ± 11.51 years, while for those with malignant lesions, it was 51.97 ± 11.95 years. This was statistically significant (t = 5.67; p < 0.001).

Among the 153 lesions, 70 were classified as BI-RADS category 3, 72 as BI-RADS category 4, and 11 as BI-RADS category 5. Several ultrasound features, including shape, orientation, margins, and calcifications, were significantly associated with the benign or malignant nature of the lesions. Four benign lesions displayed calcifications, all of which were macrocalcifications. Seven malignant lesions exhibited calcifications, including three with macrocalcifications and four with microcalcifications.

Fat necrosis in one patient showed posterior acoustic shadowing and non-parallel orientation. Three patients had ductal carcinoma in situ (DCIS), which was classified as malignant. These lesions demonstrated irregular shape, angulated margins, parallel orientation, and no distinct posterior features or associated findings. One DCIS lesion displayed microcalcifications and was located in the axillary tail.

Statistical analysis revealed that age, shape, margin, orientation and calcification were significantly associated with the nature of the lesion (Table 3). All 70 BI-RADS category 3 lesions were benign. Among the 72 BI-RADS category 4 lesions, 22 (30.56%) were malignant. All 11 BI-RADS category 5 lesions were malignant.

**Table 3:** Association of ultrasonography (USG) features with benign and malignant lesions. Statistical tests used: Chi-square test (χ²). Fisher’s exact test was applied when expected cell frequencies were low. A significance threshold of p < 0.05 was considered statistically significant.

USG features	Category	Benign (N=119)	Malignant (N=34)	p-value	Statistical test
Shape	Oval	77 (64.7%)	2 (5.9%)	<0.00001	Chi-square (χ² = 36.64)
	Irregular	42 (35.3%)	32 (94.1%)		
Margin	Circumscribed (smooth)	71 (59.7%)	2 (5.9%)	<0.00001	Chi-square (χ² = 79.03)
	Spiculated	0	16 (47.1%)		
	Ill-defined	18 (15.1%)	3 (8.8%)		
	Microlobulated	14 (11.8%)	2 (5.9%)		
	Angular	16 (13.4%)	11 (32.4%)		
Echo pattern	Hyperechoic	3 (2.5%)	0	0.54	Chi-square (χ² = 3.11)
	Hypoechoic	98 (82.4%)	31 (91.2%)		
	Isoechoic	2 (1.7%)	0		
	Heterogeneous	11 (9.2%)	3 (8.8%)		
	Complex cystic solid	5 (4.2%)	0		
Posterior acoustic features	No features	112 (94.1%)	30 (88.2%)	0.05	Chi-square (χ² = 5.9)
	Enhancement	4 (3.4%)	0		
	Shadow	3 (2.5%)	4 (11.8%)		
Calcification	Present	4 (3.4%)	7 (20.6%)	0.002	Fisher’s exact test
	Absent	115 (96.6%)	27 (79.4%)		
Orientation	Parallel	118 (99.2%)	30 (88.2%)	0.035	Fisher’s exact test
	Not parallel	1 (0.8%)	4 (11.8%)		

## Discussion

This study highlights the effectiveness of high-resolution ultrasound in distinguishing between benign and malignant breast lesions, emphasizing the importance of specific imaging features and patient demographics. Age was identified as a significant factor associated with malignancy, aligning with findings from other well-established studies. This underscores the increased risk of breast cancer with advancing age, a well-documented trend in oncology [7]. Previous research has indicated that breast cancer incidence rises significantly after the age of 40, reinforcing the need for targeted screening strategies [8].

Key ultrasound features such as shape, margins, and orientation were found to be critical in differentiating benign from malignant masses. Malignant lesions were more likely to exhibit irregular shapes and spiculated or angular margins, with 94.1% and 79.5% of malignant lesions showing these characteristics, respectively. In contrast, benign lesions were predominantly characterized by oval shapes and smooth margins (64.7% and 59.7%, respectively). These findings are consistent with prior research, such as the study by Zonderland et al., which also identified irregular contours and non-parallel orientation as strong indicators of malignancy [9]. Similarly, Costantini et al. (2007) [10] reported that non-circumscribed margins, irregular shapes, and non-parallel orientation were more frequently associated with malignant lesions. Moreover, a meta-analysis by Youk et al. (2017) [11] confirmed that spiculated margins and taller-than-wide orientation significantly increase the likelihood of malignancy in solid breast masses.

Interestingly, the internal echo pattern was not particularly useful in distinguishing between benign and malignant lesions, a finding that aligns with Stavros et al.'s work [12]. However, the presence of calcifications was a significant differentiating factor, correlating with Mordang et al.'s study [13]. Ultrasound’s ability to detect calcifications is limited compared to mammography, particularly for microcalcifications, which are more commonly associated with DCIS. A study by Berg et al. (2008) [14] suggested that while ultrasound can identify macrocalcifications and coarse calcifications, mammography remains superior in detecting clustered microcalcifications, a hallmark of early-stage malignancies.

Statistical analysis identified age, shape, margins, orientation, and calcifications as the most important descriptors for distinguishing benign from malignant nodules. The study also validated the BI-RADS classification system. All BI-RADS category 3 lesions biopsied were benign, consistent with the <2% risk of malignancy estimated by BI-RADS. Similarly, all BI-RADS category 5 lesions were malignant, aligning with the >95% risk of malignancy for this category. BI-RADS category 4 lesions in the study had a 30% risk of malignancy, which falls within the BI-RADS estimated range of 2-95%. This supports the robustness of the BI-RADS lexicon as a standardized reporting system, as noted in prior studies [15]. Additionally, Abdullah et al. (2017) highlighted that radiologists with more experience in using BI-RADS can significantly improve the accuracy of breast lesion characterization, reducing unnecessary biopsies while ensuring that suspicious lesions are appropriately evaluated [16].

Despite its strengths, the study had several limitations. First, only BI-RADS category 4 and 5 lesions, along with selected category 3 lesions, referred for biopsy, were included, leading to a higher incidence of malignant lesions in the sample. Second, advanced ultrasound techniques such as elastography were not analyzed, which could have provided additional diagnostic insights. Studies have shown that elastography can improve specificity in differentiating benign and malignant lesions by assessing tissue stiffness [17]. Finally, immunohistochemical characteristics of the lesions were not considered, which could have provided further pathological context. Immunohistochemistry plays a crucial role in subclassifying malignant lesions, guiding treatment decisions, and determining prognosis [18].

Future research should focus on integrating multiparametric ultrasound approaches, including Doppler imaging, elastography, and contrast-enhanced ultrasound, to refine the differentiation of breast lesions. Additionally, artificial intelligence (AI)-based ultrasound interpretation has shown promising results in improving diagnostic accuracy and reducing interobserver variability [19].

While ultrasound is effective, limitations suggest the need for further research incorporating advanced imaging techniques, mammographic findings, and immunohistochemical analyses for improved diagnostic accuracy. Future advancements in imaging technology and AI-driven tools could enhance early detection, reduce unnecessary biopsies, and optimize breast cancer screening, ultimately improving patient outcomes and survival rates.

## Conclusions

In conclusion, this study highlights the effectiveness of high-resolution ultrasound in differentiating benign from malignant breast lesions, with specific imaging features such as shape, margins, and orientation playing a critical role in diagnosis. Age was identified as a significant factor associated with malignancy, reinforcing the need for targeted screening strategies for women over 40. The findings also support the robustness of the BI-RADS classification system, demonstrating its accuracy in predicting malignancy risk.

However, the study acknowledges limitations, including the exclusion of advanced ultrasound techniques like elastography and the lack of immunohistochemical analysis. Future research should aim to integrate multiparametric ultrasound methods, such as elastography and contrast-enhanced ultrasound, alongside AI-based interpretation, to enhance diagnostic precision and reduce interobserver variability. Ultimately, the combination of these advanced imaging technologies holds promise for improving early detection, reducing unnecessary biopsies, and optimizing breast cancer screening, thereby improving patient outcomes and survival rates.
